# Health of New York

**Published:** 1879-06

**Authors:** 


### HEALTH OF NEW YORK.

The city of New York is capable of being made the healthiest of all the large cities of the world, and is one of the healthiest now. Much has been said about the filthiness of its streets, its underground habitations, and its crowded tenement houses, but unfortunately, the speakers and writers have not been disinterested persons, or if so, were careless in their statements, if not very ignorant of that about which they were writing. When the Hub of the Universe wishes to compare favorably against New York as to health, she gives the population and the deaths of each city, knowing at the same time that the foundation is false, for New York gives the deaths from all causes, and the regulations are so stringent that no dead body can be conveyed from the city or be buried, without official permission and faithful registry; but in Boston, the still-born are not counted, in New York they are. From time immemorial, Philadelphia stoutly has contended, and still believes, that she is larger than New York, the celebrated Frog entertained a similar opinion as to the ox, but exploded in attempting to figure it out; she claims that she has more houses than Gotham, and that the only reason why New York has a greater number of inhabitants set down to her is that the population is counted twice, because the people live on one end of the island and do business on the other, and when the census is taken, the wives are called upon at their residences, and the husbands are called upon at their places of business, thus making the returns just double.

But when Uncle Penn wants to prove that the right-angled city,with wet pavements and white door steps and green window shutters, is incomparably more healthy than the great metropolis,she believes the population statistics are most religiously true. So with the penny-a-liners and speech-makers, who have axes to grind; they compare the total number of deaths annually, with the totals given of other large cities, knowing at the time, or blissfully ignorant, that New York gives all the deaths, when some ought not, in justice. to be counted. and are not counted elsewhere

